# Prevention of Calcium Nephrolithiasis: The Influence of Diuresis on Calcium Oxalate Crystallization in Urine

**DOI:** 10.1155/2019/3234867

**Published:** 2019-03-21

**Authors:** Johannes M. Baumann, Roberto Casella

**Affiliations:** Urology, Hospital Centre Biel, Biel, Switzerland

## Abstract

A high fluid intake is still the most evidence-based measure for the prevention of idiopathic stone disease. The recommendation of current guidelines on urolithiasis to increase diuresis to 2–2.5 L/day is mainly based on a single clinical study. The present paper shows the influence of diuresis on calcium oxalate (CaOx) crystallization and especially aggregation (AGN) which can explain the initial development of Ca stones on papillary calcifications as well as stone growth in the renal pelvic system. Diuresis determines the urinary transit time (UT) through the kidney and together with the afflux of Ca and Ox the state of urinary saturation with respect to CaOx being the most frequent stone mineral. High supersaturation inducing crystallization during UT and a high urinary ion concentration interfering with the inhibition of crystal AGN by urinary macromolecules seem to be critical parameters for stone formation. Using data from the literature the influence of diuresis on these parameters is evaluated for short-term recurrent stone formers (RSF), idiopathic stone patients, and healthy controls, the latter two collectives with and without excessive oxalate ingestion. This investigation suggests that a diuresis of 2 L/day may protect from stone formation even after dietary Ox excesses and in RSF. However, in RSF with a continuously high Ca and Ox afflux into urine a permanent high diuresis is required which is difficult to sustain over 24 hours.

## 1. Introduction

Kidney stones are often accompanied by painful colic and can lead to severe renal damage or even loss of a kidney. They show in several populations over the world increasing prevalence of more than 10% with a recurrence rate up to 40% [1]. Increase of fluid intake is still the most evidence-based measure to prevent Ca nephrolithiasis in patients without metabolic anomalies [2–4]. In patients with a history of multiple stone events the addition of thiazide or citrate further reduces stone recurrence [2]. The recommendation of current guidelines on urolithiasis to increase diuresis to 2–2.5 L/day [5] is mainly based on a single clinical study where 199 Ca stone patients by randomization were divided into a group in which a high intake of water was advised and a second group without recommendation [4]. After 5 years of observation the study showed that an increase of diuresis from 1.0 to 2.6 L/day reduced stone recurrences from 27 to 12% (*p*= 0.008) and increased the stone-free interval from 25 to 39 months (*p*= 0.016). In this paper we investigate the influence of diuresis on the formation and especially the aggregation of calcium oxalate (CaOx), this being with 60% the most frequent stone compound [6].

Crystallization in urine is a complex process depending not only on the concentration of stone forming ions but also on chelators like citrate or magnesium which reduce free ionic concentration of Ca and Ox and on ionic strength diminishing the chemical activity of ions [7]. Furthermore, urinary crystals are always coated by urinary macromolecules (UM, mainly glycoproteins and some glycosaminoglycans) which essentially can influence crystallization processes [8]. Today more than 100 UM, often with not exactly known function, are described. Eleven proteins are thought to be relevant for stone formation [9]. Measurement of crystal growth showed that the growth of CaOx is too slow to allow particles within the short urinary transit time through the kidney to be able to reach dimensions being big enough to be retained in the narrow renal tubular system [10]. However freshly formed crystals by aggregation (AGN) tend to form big conglomerates. AGN is a rapid process which in urine normally is inhibited by UM [7].

Urological endoscopy showed that Ca stone formation in an initial phase starts on kidney calcifications consisting either of intratubular CaOx or Ca phosphate (CaP) aggregates (Randall's plugs) or of interstitial deposits of hydroxyapatite (HAP) broken through the epithelial layer [11]. The latter are called Randall's plaques (RPL). During the last decades much work was done to elucidate the pathogenesis of plugs and plaques and their role in stone formation [12–16]. Kidney calcifications are a frequent finding but are not always connected with stone disease. High resolution radiography of 50 consecutive sets of cadaveric kidneys showed in 57% radiographic evidence for RPL [17]. In an older study, in all kidneys of 100 randomly selected autopsies some papillary calcifications characteristic for RPL were detected [18]. However, kidney endoscopy of kidneys showed RPL which in 43% of cases were not related to stone disease [19]. Even in stone patients RPL was found to persist during decades without stone formation [20]. CaOx crystallization experiments performed in the presence of UM and HAP suggest that under special conditions UM coated RPL can give rise to stone formation by crystal AGN during crystalluria [10].

However, the question of how during phases of urinary supersaturation stones develop on kidney calcifications and how this process can be prevented is not answered definitively [21]. To get more information we studied the physiochemistry of crystallization [22] and performed crystallization experiments on urine of stone formers (SF) and healthy controls (HC).

## 2. Theoretical Bases to Understand Calcium Oxalate Crystallization in Urine and in the Kidney

Crystallization processes generally are described by the mathematical product of the chemical activities of all ions involved in the formation of a crystal, which is called activity product (AP) [7, 22]. In electrolyte containing solutions like urine the mobility of individual ions is reduced by the electrostatic forces of all ions present. The overall ionic effect can be expressed as ionic strength (IS) being half of the sum of all ions multiplied by their valence in square. Instead of the cumbersome measurement of all ions with a relevant urinary concentration, IS can be estimated multiplying urinary sodium concentration (Na) by a coefficient of 1.76. In a study of 16 SF and 12 HC the ratio of IS and urinary Na concentration at a diuresis varying from 1.3 to 3 L/day varied only about 3% with a mean of 1.76 [23]. IS reduces the chemical activity of ions by a factor f_a_ which can be calculated from IS by the following equation, z being the valence of ions:

(1) $$\begin{array}{c} -\log f_{a}=\frac{0.5\times z^{2}\times \sqrt{IS}}{1+\sqrt{IS}}. \end{array}$$

However, in urine not only is the chemical activity of ions reduced but also the free ionic concentration is diminished by the formation of highly soluble complexes of chelators like citrate with Ca or magnesium with Ox. For the calculation of AP from the molar concentration of Ca (Ca_m_) and of Ox (Ox_m_) by (2) chelating effects are considered introducing the factor f_Ca_ for the free fraction of Ca and f_Ox_ for that one of Ox:

(2) $$\begin{array}{c} AP={Ca}_{m}\times {Ox}_{m}\times f_{Ca}\times f_{Ox}\times {f_{a}}^{2}. \end{array}$$

Crystallization starts when a solution is supersaturated with respect to a solid phase or in other words when the AP in the solution exceeds the solubility product (SP_a_) the AP being found after equilibration of a solution with a crystal mass in excess. SP_a_ is a characteristic for each sort of crystals and apart from temperature independent from all other conditions in a solution. Supersaturation which is the driving force for crystallization can be expressed either by the ratio of the actual activity product AP found in a solution and SP_a_ [7] or by the ratio of the molar concentration products of the crystal forming compounds before (CP_m_) and after equilibration of a solution with the corresponding crystals in excess (SP_m_) [24]. A ratio above 1.0 indicates supersaturation; at a ratio below 1.0 the solution is undersaturated. Since the ratio of Ca and Ox concentration in urine is about 20:1 and the chelation of Ca and of Ox about 5:2 changes of urinary Ox concentrations have a greater influence on the state of CaOx saturation than changes of Ca concentration. From Ca, Ox, phosphate, citrate, magnesium, and pH which are thought to be relevant for stone formation only the urinary Ox concentration showed a significant correlation (*p*<0.001) with urinary saturation with respect to CaOx which was determined in 76 urine specimens of SF by equilibration experiments [24]. Furthermore, in a clinical study of 53 SF the extent of crystalluria and even the rate of stone recurrence were highly related to the daily urinary excretion of Ox but not of Ca [25].

At the start of crystallization initially only small crystals composed of a few molecules are formed which rapidly dissolve. To become stable particles, crystals must grow to a size where the free energy associated with the liquid-solid phase change exceeds the energy necessary to build up crystal surfaces against surface tension [22]. The critical supersaturation inducing visible crystal formation is called formation product which can be expressed as AP or CP_m_. To form stable particles at a given CP_m_ a critical time called induction time (IT, min) is required. IT decreases with increasing CP_m_ and can be calculated for CaOx by (3) from Ca_m_ and Ox_m_ and an induction constant (k_I_, 16.7 min mM^2^). In (3) the original formula based on M and seconds [22] is transformed to mM^2^ and minutes:

(3) $$\begin{array}{c} IT=\frac{k_{I}}{{\left({Ca}_{m}\times {Ox}_{m}\right)}^{1.66}}. \end{array}$$

After formation, crystals tend to aggregate to large conglomerates by the attraction of the Van der Waals forces (VWF) which have an activity being limited to a few nanometers [26]. Therefore, for AGN particles must collide by diffusion or sedimentation. Under physiological conditions this mainly occurs by sedimentation where collision rate increases with increasing particle concentration [27]. However, in urine crystals are always coated by UM which due to their anionic groups like carboxyglutamic acid, phosphate, or sialic acid have an electronegative charge [8]. VWF are thus counteracted by the electrostatic repulsion exerted by the identically charged UM coats. The electrical surface charge of UM is with about -15 mV at the limit for \ definite inhibition of AGN or suspension stability [28]. Therefore, in urine AGN occurs always with some delay which in relation to the rapid urinary passage through the kidney may be decisive for stone formation.

In recurrent stone formers CaOx and CaP nucleation can take place at the end of the descending limb of the loop of Henle [15, 29]. However, in most patients CaOx crystallization seems to start at the end of the collecting ducts where urinary passage is only in the order of few seconds [10]. Urinary transit time (UT) through the renal pelvic system on the other hand endures several minutes and is essentially influenced by diuresis. UT (minutes) can be calculated by (4) from the twofold renal pelvic volume (V_P_, varying from 5 to 9 ml), diuresis (D, L/24 h), and a constant k_T_ (1.44 min L h^−1^ ml^−1^) which transforms hours to minutes and L to mL:

(4) $$\begin{array}{c} UT=\frac{2V_{P}\times k_{T}}{D}. \end{array}$$

## 3. Experimental Investigation for a Better Understanding of the Formation of Calcium Stones

**Figure 1:**
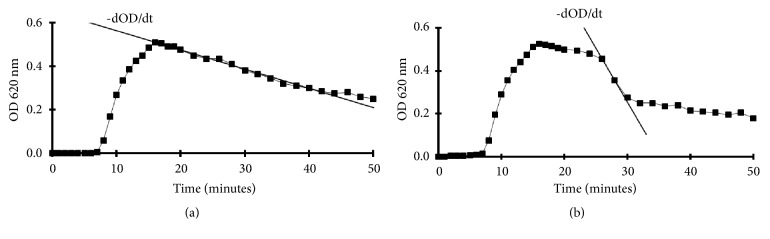
Crystallization curves in urine obtained by Ox titration. (a) Urine with inhibition of AGN demonstrated by slow decrease of optical density (-dOD/d). (b) Urine with massive AGN indicated by a phase of rapid -dOD/dt.

CaOx crystallization was studied by Ox titration of urine and control solutions in a spectrophotometer [30]. By the Ox titration we tried to imitate continuously increasing urinary supersaturation as it may occur after dietary Ox excesses. Furthermore, the effect of Randall's plaques (RPL) was simulated repeating the tests in the presence of HAP crystals. Crystal formation was monitored by a continuous measurement of optical density (OD) which at 620 nm mainly reflects particle concentration [31]. After a critical Ox addition which is a measure of the metastability of a solution or urine OD rapidly increased indicating massive crystal formation (Figures 1(a) and 1(b)). At the end of titration magnetic stirring was stopped and the further course of OD was followed.

Two typical crystallization curves were found as shown in Figures 1(a) and 1(b). One type (Figure 1(a)) showed after titration a continuous slow OD decrease characteristic for sedimentation of single crystals or for an inhibition of AGN, respectively. The other type (Figure 1(b)) showed after an initial phase of slow OD decrease varying from 7 to 35 minutes a rapid OD decline indicating crystal aggregation. Since OD mainly reflects particle concentration the rapid OD decrease represents increased particle clearing in the spectrophotometer. This high clearing is based on accelerated sedimentation which increases with particle diameters in square [27] as well as on the diminution of crystal concentration by their integration into few large aggregates.

CaOx AGN in urine was studied under different experimental conditions. In a first study urine was collected from 30 SF and 30 HC and immediately frozen [30]. To obtain comparable results Na concentration essential for ionic strength was always adapted to an identical value of 100 mM before performing crystallization tests. Under this condition urine showed in 19 of 30 HC and in 10 of 30 SF inhibition of CaOx AGN (*p*< 0.05). Interestingly this inhibition was abolished in all urine specimens when tests were repeated after the addition of HAP crystals. AGN started in urine with HAP addition after a delay of 6 to 13 minutes within an average residence time of urine in the renal pelvis of 12 minutes [30]. AGN also occurred in all UM solutions which were obtained either by dialysis of urine by a hemofilter procedure [28] or by Ca phosphate precipitation as described below. On the other hand, in 14 of 15 freshly voided urine specimens of HC even after Ox addition of 1.5 mM with massive crystal formation no AGN could be observed [32]. However, after the addition of 0.05 mg/mL HAP and repeating the crystallization test the inhibition of AGN was abolished in 8 of the 14 urine specimens. These 8 urine specimens showed a significantly higher Na concentration than those where inhibition persisted (125 ± 17 vs. 68 ± 23,* p* < 0.01). A relative high Na concentration as applied in the first study and HAP crystals thus seem to be important promoters of CaOx AGN. When the test with HAP addition was repeated after dilution of the urine to 50% and an adaption of Ca^+2^ and pH to original values inhibition was fully restored. Also in a study of urine dilution by increasing fluid intake which was performed in 16 SF and 12 HC an inverse relationship between changes of urine volume and CaOx AGN (*p*= 0.004) was found [23]. Since urinary inhibitor capacity especially with respect to AGN is very high dilution remains an appropriate measure to prevent AGN despite a reduction of inhibitor concentration. The clinical implication of a reduced renal papillary density which was observed by computer tomography in 25 SF after at least 12 month of hydration therapy deserves further evaluation [33].

The promotion of AGN by Na can be explained by its contribution of more than 50% to urinary ionic strength (IS) [23]. At high IS the extension of electronegative surface potentials responsible for the inhibition of AGN is compressed to a few nanometers by the accumulation of cations like Na on particle surfaces [26]. Under these conditions the Van der Waals forces despite their limited reach of action can overwhelm the electrostatic repulsion exerted by the identically charged UM coats of the crystals.

**Figure 2:**
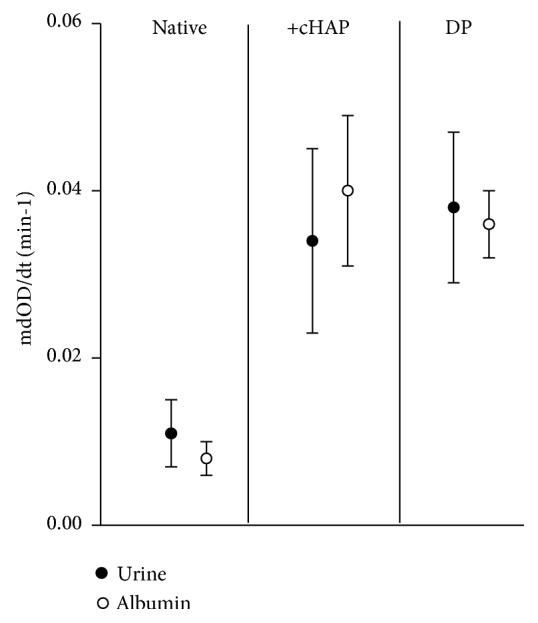
Maximal decrease of optical density (mOD/dt) in crystallization tests performed in urine and albumin solution (AS, 20 mg/ml) without (native) and after the addition of UM coated hydroxyapatite (+cHAP, 0.05 mg/ml) and in dissolved Ca phosphate precipitates (DP) from urine and AS.

Experiments performed with albumin which is one of the most abundant UM and the major compound of the stone matrix [8] gave some insight into the promotion of AGN by HAP. Several UM have a high affinity to Ca phosphate (CaP) [34] which can be used for their isolation [35]. To this purpose CaP precipitation was induced in urine and in a solution of albumin in a high physiological concentration (AS, 20 mg/mL) at pH 7.0 by the addition of P to urine and of Ca and P to AS. Afterwards the precipitates were dissolved at pH 5.0 and after adaption of Ca^+^, Na, and pH to urinary values CaOx crystallization tests were performed. Maximal OD decreases observed in these experiments after the end of Ox titration are compared in Figure 2 with those measured in urine and in AS with and without previous addition of HAP [32].

Whereas maximal OD decrease in urine and AS without HAP was low (indicating inhibition of AGN) it showed in the presence of HAP a massive increase characteristic for AGN. AGN also occurred in the dissolved CaP precipitates (DP) of urine and AS without HAP addition. Therefore, the promotion of CaOx AGN by HAP seems not to be based on epitaxy but on the ability of HAP to concentrate UM by adsorption. Such a concentration also seems to occur on filter membranes since urine showed after dialysis as mentioned above in all crystallization tests massive AGN.

UM like albumin, osteopontin, and the Tamm-Horsfall glycoprotein, the latter two also important for stone formation, have at high concentration a tendency to self AGN [8]. Adsorption on surfaces creates such critical concentrations. This could be demonstrated by the measurement of particle size distribution by a Zetasizer in AS before and after temporary adsorption of the albumin on CaP [35].

**Figure 3:**
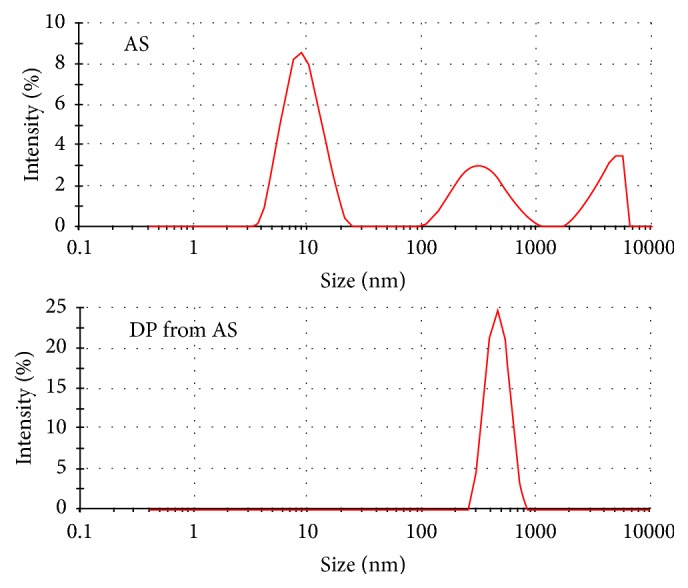
Particle size distribution in albumin solution (AS, 20 mg/ml) and in dissolved Ca phosphate precipitate of AS (DP from AS).

In AS apart from a main peak at 10 nm characteristic for single albumin molecules further smaller peaks indicating aggregates were observed (Figure 3). On the other hand, in the dissolved CaP precipitate of AS all albumin which temporarily had been adsorbed on CaP was found collected in massive aggregates within a single peak around 420 nm. Such large particles can bridge zones of electrostatic repulsion and connect urinary crystals by hydrophobic binding to their UM coats [35].

Histological analysis, immunohistochemistry, and infrared spectroscopy of Randall's plaques (RPL) with an adherent CaOx stone gave further evidence for the importance of self-aggregating UM in stone formation [11]. The plaques consisted of HAP deposits within an osteopontin matrix whereas the adherent stone mainly contained CaOx crystals embedded in the Tamm-Horsfall glycoprotein. Stone formation on RPL thus occurred at the interface of UM which have a high tendency to self AGN but not by a direct contact between HAP and CaOx crystals. Epitaxy between HAP and CaOx, which for a long time was thought to be important for stone formation, is hindered by the UM layers of the crystals. Promotion of CaOx crystallization by UM [36] is at least in idiopathic Ca nephrolithiasis not very probable since in our experiments the critical Ox addition for crystallization was always higher in urine and in UM containing mediums than in the UM free control solution [30]. Therefore, the apposition of CaOx crystals to RPL and already existing stones mainly seems to be based on AGN during crystalluria [7, 37, 38].

## 4. The Influence of Diuresis on Urinary Risk Factors for Calcium Stone Formation

Theoretical considerations and experimental data suggest that stone formation mainly depends on the afflux of stone forming ions, on urinary ionic strength which can be estimated from urinary Na concentration, and on diuresis which determines ionic concentration in urine and urinary transit time through the kidney. Representative information about these factors in SF and HC was found in three studies with at least 30 participants [4, 38, 39]. Data from a fourth study of 6 patients with short-term stone recurrence (RSF, at least 2 stone episodes per year) were included [15]. Furthermore, maximal urinary excretion of Ca and Ox per hour which was observed after the ingestion of vegetables (spinach and rhubarb) equivalent to 1200 mg Ox in 11 SF and 10 HC [40] and of 100 g chocolate in 6 HC [41] is presented. Results of the different studies were compiled in the 5 groups SF, RSF, and HC as well as SF and HC after dietary Ox load (SF +OL and HC +OL). For each group the means of the values of the individual studies are indicated in Table 1.

From the mean urine volume and afflux of Ca and Ox in urine per day or hour, respectively, we tried to estimate the state of urinary saturation (SS) with respect to CaOx which can be expected in the different groups at varying states of diuresis. Since in several studies information about SS was lacking and no equilibration experiments were performed, molar concentration products (CP_m_) and molar solubility products (SP_m_ ) were calculated for a physiological range of diuresis (0.75–2.5 L/day). SP_m_ was obtained from the known thermodynamic solubility product (SP_a_, 0.0036 mM^2^) [42] by the following equation which was derived from (2):

(5) $$\begin{array}{c} {SP}_{m}=\frac{{SP}_{a}}{f_{Ca}\times f_{Ox}\times {f_{a}}^{2}}. \end{array}$$

**Figure 4:**
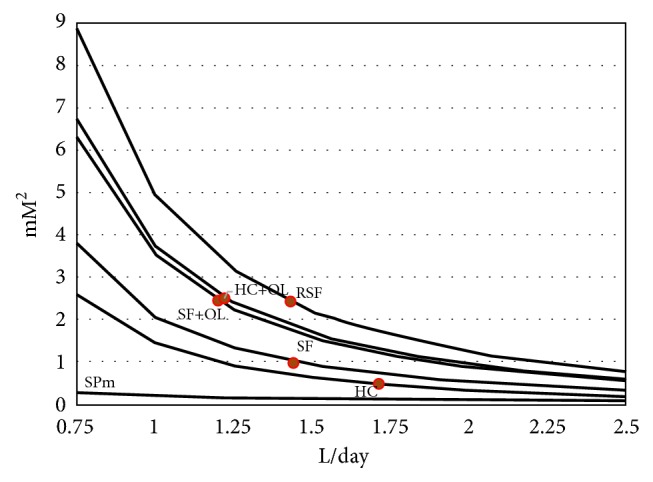
Molar urinary concentration products of Ca times Ox (CP_m_, mM^2^) in stone formers (SF) and healthy controls (HC), with dietary Ox load (+OL) and in recurrent SF (RSF) compared to molar solubility products of CaOx (SP_m_) at different states of diuresis (L/day): CP_m_ at the average diuresis in the group (o).

Free urinary Ca fraction (f_Ca_) was taken from a study of 60 SF and 60 HC where f_Ca_ in SF was 0.54 ± 0.01 and in HC 0.49 ± 0.01 [39]. For free Ox fraction (f_Ox_) a value of 0.8 was assumed. For the calculation of the ion activity coefficient (f_a_) by (1) ionic strength (IS) was estimated multiplying Na concentrations by a factor 1.76 as explained above. CP_m_ and SP_m_ are compared in Figure 4 at different states of diuresis.

**Figure 5:**
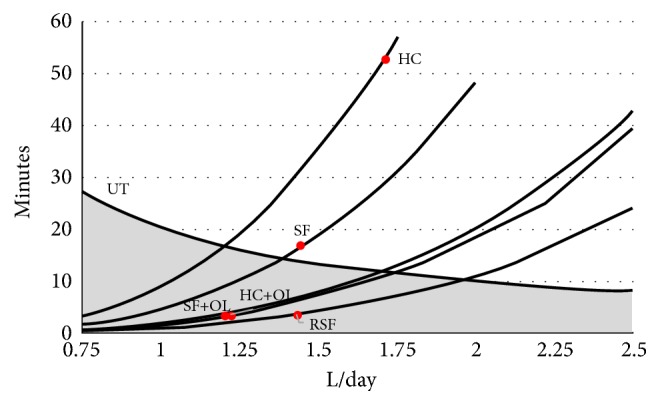
Induction time of crystallization (IT, minutes) in SF and HC, with dietary Ox load (+OL) and in RSF compared to urinary transit time (UT) through the renal pelvic system (volume 7 mL) at different states of diuresis (L/day): IT at the average diuresis in the group (o), crystallization occurring within the kidney (shaded area).

The figure demonstrates that urine generally is supersaturated with respect to CaOx since in all groups and at all states of diuresis CP_m_ always is higher than SP_m_. Even in HC and at high diuresis CP_m_ hardly can be brought below SP_m_. The minimal change of SP_m_ with variations of diuresis compared to that one of CP_m_ shows that CP_m_ mainly determines SS which can be expressed by the ratio of CP_m_ and SP_m_. This is confirmed by a study performed in 30 SF and 30 HC where a linear correlation (r= 0.92) between CP_m_ and computer calculated SS was found [43]. In RSF and in SF and HC after excessive Ox ingestion a high CP_m_ is observed which especially at low diuresis extremely increases. Such a high CP_m_ induces rapid crystallization with a very short induction time (IT) which can be calculated from CP_m_ by (3). IT is compared in Figure 5 with urinary transit time through the renal pelvic system (UT).

Since the renal passage of crystals due to fluid drag [44] and viscous binding of crystals to epithelial layers [45] can endure much longer than UT, it is important for stone prevention that IT exceeds UT or in other words that crystallization exclusively occurs after urine has left the urinary collecting system of the kidney. Figure 5 shows that this is achieved by a diuresis above 2 L/day even in RSF and in SF and HC after excessive Ox ingestion. Retained crystals otherwise may have the time for AGN which in the presence of HAP is in the order of 6–13 minutes [30]. Furthermore, a diuresis above 1.75 L/day directly may prevent crystal AGN by decreasing ionic strength.

**Figure 6:**
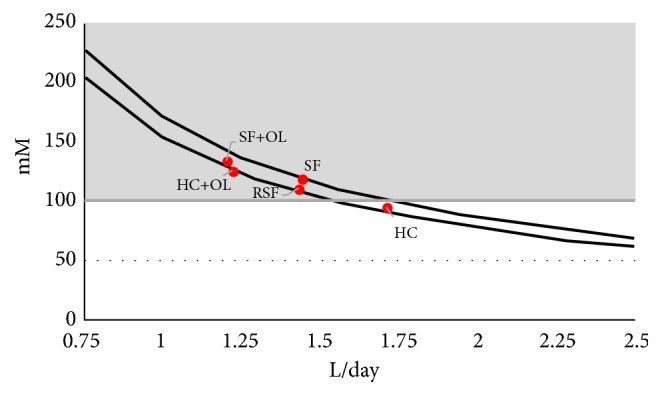
Urinary sodium concentration (Na, mM) in SF and HC, with dietary Ox load (+OL) and in RSF at different states of diuresis (L/day) and critical Na concentration for AGN (100 mM): Na concentration at the average diuresis in the group (o), risk of AGN (shaded area).

Figure 6 shows that at a diuresis above 1.75 L/day urinary Na concentration being an indicator for IS generally falls below the 100 mM found in our experiments to be critical for HAP induced AGN. The minimal difference of Na concentration between SF and HC when plotted versus diuresis demonstrates that at least in the reviewed studies overconsumption of Na is not a risk factor for stone formation [46]. Urinary Na concentration in these cases mainly seems to depend on diuresis.

## 5. Summary and Conclusions

Clinical and experimental investigation shows that Ca and Ox afflux to urine and diuresis are important factors for Ca stone formation. Kidney calcifications being an important source of stones are a frequent finding even without stone disease. CaOx crystals too often are observed in urine of SF and HC and harmless if crystal formation occurs outside of the kidney and ionic concentration of urine remains below a value that is critical for AGN. This can as Figures 5 and 6 show be achieved by a high diuresis being adapted to the afflux of stone material. In RSF with their permanent high afflux of Ca and Ox this would require a continuously high fluid intake which especially overnight can hardly be sustained. These patients are known to suffer despite some medical treatment from a very frequent stone recurrence. Several genetic disorders inducing hypercalciuria and hyperoxaluria were found to be associated with the formation of CaOx stones [47]. However, the majority of SF show no genetic or metabolic anomalies and only a low recurrence rate. A review of 31 representative publications revealed in these idiopathic called SF a stone frequency of 0.1–0.15 per patient and year or in other words an interval of about 8 years between two stone events [48]. Stone formation in idiopathic SF thus seems to be more the result of a coincidence of noisy factors than a real disease. A dangerous constellation is excessive ingestion of Ox rich food in combination with a poor fluid intake. This constellation is as Figure 5 shows also possible in HC and could explain the high prevalence of stone formation observed in several populations over the world [1]. A high fluid intake which guarantees a diuresis of 2 L/day seems together with some dietary Ox and Na restriction to be an appropriate measure to prevent idiopathic Ca nephrolithiasis. Determining the concentration product of Ca and Ox and the concentration of Na in 24-hour urine may help to get a personalized recommendation for an adequate diuresis. Considering excretion profiles after oral Ox loads [41] a high fluid intake should be advised especially after Ox rich meals.

## Figures and Tables

**Table 1:** Afflux of important components for stone formation in urine of stone formers and healthy controls.

Reference	Participants	Volume	Na	Ca	Ox
	Number	L/d	mM/d	mM/d	mM/d

*Stone formers*					

[39]	60	1.73	193	8.15	0.23

[38]	86	1.952	170	6.3	0.37

[4]	99	1.068	158	6.1	0.32

[4]	100	1.008	162	6.65	0.32

Mean		*1.439*	*171*	*6.8*	*0.31*

*Recurrent stone formers*					

[15]	6	*1.43*	*159*	*9.05*	*0.55*

*Healthy controls*					

[39]	60	1.56	172	5.96	0.22

[38]	36	1.868	155	4.86	0.31

Mean		*1.714*	*163*	*5.41*	*0.27*

		ml/h	mM/h	*μ*M/h	*μ*M/h

*Stone formers after dietary Ox load*					

[40]	11	*50*	*6.71*	*121*	*51*

*Healthy controls after dietary Ox load*					

[40]	10	51	6.42	113	48

[41]	6			215	32

Mean		*51*	*6.42*	*164*	*40*
